# Detecting pre-death grief in family caregivers of persons with dementia: measurement equivalence of the Mandarin-Chinese version of Marwit-Meuser caregiver grief inventory

**DOI:** 10.1186/s12877-018-0804-5

**Published:** 2018-05-11

**Authors:** Tau Ming Liew, Philip Yap, Nan Luo, Soo Boon Hia, Gerald Choon-Huat Koh, Bee Choo Tai

**Affiliations:** 1 Department of Geriatric Psychiatry, Institute of Mental Health, 10 Buangkok View, Singapore, Singapore; 2 Psychotherapy Service, Institute of Mental Health, Singapore, Singapore; 3 Saw Swee Hock School of Public Health, National University of Singapore, Singapore, Singapore; 4 Department of Geriatric Medicine, Khoo Teck Puat Hospital, Singapore, Singapore; 5 Geriatric Education and Research Institute, Singapore, Singapore

**Keywords:** Pre-death grief, Family caregivers, Dementia, Chinese, Marwit-Meuser caregiver grief inventory, Measurement equivalence

## Abstract

**Background:**

Pre-death grief (PDG) is a key challenge faced by caregivers of persons with dementia (PWD). Marwit-Meuser Caregiver Grief Inventory (MM-CGI) and its abbreviated MM-CGI-Short-Form (MM-CGI-SF) are among the few empirically-developed scales that detect PDG. However, they have not had a Mandarin-Chinese version even though Chinese-speaking populations have among the largest number of PWD. We produced a Mandarin-Chinese version of MM-CGI and evaluated whether it had equivalent scores and similar psychometric properties to the English version.

**Methods:**

We produced the Chinese MM-CGI through the methods of forward-backward translation and cognitive debriefing. Then, we recruited family caregivers of PWD (*n* = 394) to complete either the Chinese (*n* = 103) or English (*n* = 291) version. The two versions were compared in their score-difference (adjusting for potential confounders using multiple linear regression), internal-consistency reliability (using Cronbach’s α) and test-retest reliability (using intraclass correlation-coefficient), known-group validity (based on the relationship with the PWD and stage of dementia) and construct validity (using Spearman’s correlation-coefficient).

**Results:**

The two versions showed similar mean scores, with the adjusted score-difference of 1.2 (90% CI -5.6 to 7.9) for MM-CGI and − 0.4 (90% CI -2.9 to 2.1) for MM-CGI-SF. The 90% CI for adjusted score-difference fell within predefined equivalence-margin (±8 for MM-CGI and ± 3 for MM-CGI-SF) and indicated equivalence of the scores. The two versions also demonstrated similar characteristics in reliability and validity.

**Conclusions:**

The Chinese MM-CGI opens the way for PDG assessment and intervention among Chinese-speaking caregivers. Establishing its measurement equivalence with the English version paves the way for cross-cultural research on PDG in dementia caregiving.

**Electronic supplementary material:**

The online version of this article (10.1186/s12877-018-0804-5) contains supplementary material, which is available to authorized users.

## Background

With the rising prevalence of dementia globally [1], more family members will inevitably be drawn into the care of their relatives with dementia. Providing care to a person with dementia (PWD) is often protracted, stressful [2] and not uncommonly associated with physical and emotional burden [2–4]. Caregiver burden, in turn, has been shown to produce less desirable outcomes such as premature nursing home placement of the PWD [5] and greater risk of mortality in the PWD [6]. For this reason, much research has targeted caregiver burden and the appropriate interventions to alleviate burden [3]. However, the loss and grief that caregivers often experience while providing care is less commonly recognised.

Caregivers of PWD may begin their bereavement long before the physical death of the PWD [7]. The caregivers’ responses to perceived losses in the pre-death context – which we refer to as pre-death grief (PDG) – include the anticipation of future loss related to the physical death of PWD and the mourning of present loss related to the psychological death of PWD (a unique phenomenon in dementia whereby the PWD is still physically present but emotionally disconnected from the caregiver) [8]. In a recent systematic review from the British Journal of Psychiatry [9], PDG has been highlighted as a key challenge faced by caregivers, with the loss of emotional bond with PWD accentuating caregivers’ unmet needs for nurture, companionship and emotional security. PDG has also been described as the central experience of spousal caregivers of PWD in another recent systematic review of the qualitative studies [10]. The prevalence of PDG has been reported in a few studies and ranged between 47 and 86% [8, 11].

PDG in dementia caregiving can be more complicated than the usual experience of loss and grief [8, 12]. The protracted trajectory of dementia prolongs the caregivers’ sense of uncertainty about the loss. During this protracted period of uncertainty, the communication with the PWD also becomes disrupted and there is no longer any opportunity to reaffirm the relationship. To make the matter worse, such experience of grief before the physical death of PWD may not be socially sanctioned, leaving the caregivers with the sense of isolation as they find it difficult to share their feelings with the people around them. When the experience of loss and grief is not recognized, caregivers may have difficulty accepting any changes that have occurred in the PWD and may attempt to fight the inevitable decline in the PWD. To that end, they may be more inclined to cope using control-based strategies, become more paternalistic in their communication with the PWD and adopt more authoritarian decision-making styles in caregiving. Eventually, this can affect the well-being of both the PWD and the caregivers, with the PWD feeling the sense of loss of autonomy and the caregivers feeling more helpless over what they cannot control [13]. Not surprisingly, high levels of PDG have been associated with caregiver burden [14] and depression [11], and increase the risk of caregivers considering nursing home placement for the PWD [15]. However, caregivers with high PDG may not be easily recognised in clinical practice because PDG is commonly disenfranchised [12] and not readily talked about by caregivers. They may also be missed by other routinely-used scales such as the caregiver burden scale as demonstrated by a recent study [8], thus indicating the need for a scale that specifically measures PDG.

The Marwit-Meuser Caregiver Grief Inventory (MM-CGI) [16] and its abbreviated MM-CGI-Short-Form (MM-CGI-SF) [17] are among the few empirically-developed scales that measure PDG [11, 12]. However, it has not had a Mandarin-Chinese version even though the Mandarin-Chinese language is among the most widely-used language worldwide [18], and countries with Chinese-speaking populations have among the largest number of PWD (with 9.5 million in China alone) [1]. Although there was a recent study that validated the Chinese MM-CGI-SF, it only focused on the Cantonese-Chinese version [19]. Cantonese-Chinese is a dialect of the Chinese language that is used in only 5% of the Chinese-speaking community [18], in contrast to the Mandarin-Chinese which is used in 70% of the community [18] and is recognised as the national language in countries such as China and Taiwan. While the Cantonese-Chinese and Mandarin-Chinese share similarities in the written characters, they are distinct and may not be mutually intelligible.

In this study, we produced the Mandarin-Chinese version of MM-CGI for use among Chinese-speaking caregivers. As the language of a scale can have prominent influence [20] on individuals’ responses to culturally-sensitive questions (such as those related to grief) [21], we are uncertain whether the new Mandarin-Chinese version will be equivalent in its measurement scores to those of the original English version. This is an important issue for future research that endeavours to compare the scores between the Chinese- and English-speaking populations in cross-cultural studies, or pool data from the two language versions to improve statistical power and representativeness of a study. As such, we sought to evaluate whether the Chinese versions of MM-CGI and its abbreviated MM-CGI-SF are equivalent in their measurement scores to the original English version. As a secondary aim, we also sought to evaluate whether the Chinese and English versions demonstrate similar patterns in reliability and validity assessment.

## Methods

### Development of the Chinese version of Marwit-Meuser caregiver grief inventory

We produced the Mandarin-Chinese version of MM-CGI [16] by applying the methods which have been recommended by the International Society for Pharmacoeconomics and Outcomes Research (ISPOR) [22]. Briefly, the methods included forward translation, back-translation and cognitive debriefing. The details of each step are described in the paragraphs below.

In the first step (forward translation), two native speakers of Chinese language (SJCL and SBH) independently translated the MM-CGI into Chinese versions. Both copies of the forward translation were reconciled by an independent native speaker of Chinese language (TML), in discussion with the two forward translators whenever necessary.

In the second step (back-translation), two other persons translated the reconciled Chinese version back to the English language (HLS and DLLC). These two back-translators were native speakers of English language who had not seen the original MM-CGI. Thereafter, two independent reviewers (KST and TML) compared the back-translations against the original English version of MM-CGI to resolve any discrepancies.

The final step, cognitive debriefing, involves conducting qualitative interviews on a small group of people from the target language to assess the level of comprehensibility and cognitive equivalence of the translation, and to identify any items that may potentially cause confusion [22]. We conducted the cognitive debriefings on three professional caregivers and three family caregivers – the three professional caregivers (YH, HL and CL) were nurses who were working in a geriatric psychiatry ward and had at least three years of experience in the routine care of PWD; while the three family caregivers were children caregivers who accompanied their relatives with dementia for routine clinical care. During the cognitive debriefing, we asked the caregivers to rephrase each item in MM-CGI to assess their understanding. We also encouraged caregivers to highlight any items that were difficult to understand. Then, two researchers (BIY and TML) independently reviewed the transcripts of the verbatim responses. They proposed amendments when both of them agreed that an item was unclear or was interpreted differently from the original intent. Any disagreement was resolved by referring to a third researcher (PY). Any proposed amendment also required the review and agreement by the third researcher (PY).

The final version of Chinese MM-CGI is presented in Additional file 1.

### Participants and procedures

Upon producing the Chinese MM-CGI, we consecutively-sampled caregivers from two tertiary hospitals in the North-East of Singapore to complete the Chinese version of MM-CGI. These are caregivers who accompanied the PWD to the dementia care services and fulfilled the following criteria: (i) spouses or children of PWD; (ii) caring for PWD who is residing in the community; (iii) age ≥ 21 years; and (iv) able to read in Chinese. The recruitment criteria were predicated on our definition of family caregivers, which includes family members who are involved in the care of the PWD either directly or indirectly [23] – hence, we considered the act of accompanying the PWD to the dementia care services as an evidence of indirect involvement with the care of the PWD, which qualifies a person as a family caregiver. At the point of recruitment, the participants completed on-site the Chinese version of MM-CGI, together with a caregiver burden scale (Zarit Burden Interview, ZBI) and a depression scale (Centre for Epidemiologic Studies-Depression Scale, CES-D). One week later, the participants were reminded through phone to complete a second questionnaire at home and to mail back the completed questionnaire to us, for the purpose of assessing test-retest reliability.

We also needed participants who have completed the English MM-CGI to allow comparisons between the Chinese and English versions of MM-CGI. To that end, we included the dataset from our separate study [8] which recruited 300 English-speaking caregivers to complete the English MM-CGI. This other study was a replica of the current study (sharing the same recruitment site, study period, inclusion criteria and study procedures), with the only difference in the language of administration (the separate study was in English, in contrast to the current study which was in Chinese). The details of this other study are also available at [8].

Altogether, we recruited 403 participants, with 103 completing the Chinese version and 300 (from our separate study) [8] completing the English version. We limited our analysis to one caregiver for each PWD in the study to avoid between-participant correlation among those from the same household and consequently, excluded 9 participants from the English version because they were from the same household as some of those who completed the Chinese version. Hence, the final analysis only included 394 participants (103 in the Chinese version and 291 in the English version), with one caregiver for each PWD in the study. Following the initial questionnaire, 60 % of the participants mailed back a second set of the questionnaire for assessment of test-retest reliability (61.2% among those who completed the Chinese version and 59.8% among the English version). Median time of completing the second questionnaire was 11 days, with an inter-quartile range of 8–13.

The Domain Specific Review Board of Singapore granted ethical approval for the study.

### Measures

The Marwit-Meuser Caregiver Grief Inventory (MM-CGI) is among the few PDG scales that has been empirically-developed, following extensive focus-group interviews with caregivers of PWD [16]. It has 50 items that assess PDG on 5-point Likert scales and produces a maximum score of 250. The original validation study [16] also revealed 3 dimensions of loss in the exploratory factor analysis – *Personal Sacrifice Burden*, *Heartfelt Sadness and Longing,* and *Worry and Felt Isolation*. The *Personal Sacrifice Burden* dimension captures the personal aspect of losses experienced by caregivers (such as loss of personal freedom, sleep and physical health), the *Heartfelt Sadness and Longing* dimension captures the traditional concept of grief (that is, one’s intrapersonal reactions to lost relationship) and the *Worry and Felt Isolation* dimension captures the feelings of losing connection with others and the worry about future losses. These 3 dimensions of loss are not dissimilar to the 3 aspects of loss previously conceptualized by Dempsey and Baago [24], namely the loss of personal identity, loss of the PWD, and symbolic loss of the ideal. MM-CGI also has a shorter version, the MM-CGI-Short Form (MM-CGI-SF), which uses only the 18 most representative items for ease of administration [17]. MM-CGI-SF is also rated on 5-point Likert scales and has a maximum score of 90. Both MM-CGI and MM-CGI-SF have been validated in Singapore [8].

The Zarit Burden Interview (ZBI) is a 22-item scale that measures the perceived burden experienced by caregivers of older persons. It is scored on 5-point Likert scales, with the total score ranging from 0 to 88. According to the original test instructions [25], a score range of 21–40 indicates mild burden, while 41–60 indicates moderate burden and 61–88 indicates high burden. In a more recent study [26], scores ≥34 were reported to indicate significant burden that may require clinical attention. ZBI has been shown to contain 5 domains – *Burden in the Relationship*, *Emotional Well-being*, *Social and Family Life*, *Finances*, and *Loss of Control over one’s life* [27]. The Centre for Epidemiologic Studies-Depression Scale (CES-D) comprises 20 items which measure depressive symptomatology on 4-point Likert scales. The total score ranges from 0 to 60, with scores ≥16 indicating significant depression [28, 29]. CES-D contains 4 domains – *Depressed affect*, *Positive Affect*, *Somatic Symptoms*, and *Interpersonal Problems*. ZBI and CES-D have previously been validated in Singapore [30, 31].

Key information on the caregiver and PWD was also captured in the study. The information was based on self-reports by the caregivers or obtained from the medical records when the caregivers were uncertain. Caregiver data included age, gender, ethnicity, marital status, employment status, educational attainment, relationship with PWD, whether the caregiver stayed with the PWD, duration of caregiving, frequency of contact with the PWD and primary caregiving role. Data relating to the PWD included age, gender, duration of dementia diagnosis and stage of dementia. The stage of dementia was captured using a brief measure based on the descriptions of the three dementia severities described in the revised third edition of Diagnostic and Statistical Manual of Mental Disorders (DSM-III-R) [32]. From the three options, participants chose the description that best described the PWD – still capable of independent living (mild stage), needs some assistance with daily living (moderate stage), or needs round-the-clock supervision (severe stage). This brief measure was previously shown to have reasonable agreement (kappa 0.56–0.6) [33, 34] with Clinical Dementia Rating Scale, which is one of the most commonly-used scale to stage dementia [35, 36]. This brief measure is also nearly identical to the re-introduced dementia severity in DSM-5 [37].

### Statistical analyses

We computed the score-difference between the language versions using multiple linear regression, adjusting for potential confounding variables (relationship with PWD, stage of dementia and the baseline variables which were significantly different between the two language versions). Relationship with the PWD and stage of dementia were included in the statistical adjustment because these two variables have been reported in the literature to be predictors of PDG [11, 38].

Based on the approach to evaluate therapeutic equivalence in clinical trials, we defined a priori that equivalence in the scores of the Chinese and English versions would be declared if the 90% confidence interval (CI) of the adjusted score-difference fell within a predefined margin of equivalence. The use of 90% CI is in line with internationally-accepted practice of evaluating bioequivalence in clinical trials [39, 40], and corresponds to conducting two one-sided tests of hypothesis at the 5% level of significance [41]. The margin of equivalence refers to a predefined range of values that would be considered too small to be clinically meaningful, and any differences which are lesser than these predefined values would be small enough to reasonably claim equivalence. In this study, we predefined the margin of equivalence as approximately ±5% of the reported average values of MM-CGI and MM-CGI-SF from the original publications [16, 17]. This translated into margins of equivalence of ±8 for MM-CGI and ± 3 for MM-CGI-SF. The respective margins of equivalence for the subscales of MM-CGI and MM-CGI-SF are also presented in Table 2. Should the 90% CI fall outside of the ±5% margin, we will still consider the presence of possible equivalence if the confidence interval does not exceed the less-stringent ±10% margin (which is ±16 for MM-CGI and ± 6 for MM-CGI-SF). On the other hand, should the 90% CI fall entirely outside of the 10% margin, we will declare that there are significant differences in the scores between the Chinese and English versions.

Additionally, we also compared the two language versions with respect to their reliability and validity – we expected that the 95% CI of all the reliability indices should fall within the range of ≥0.70 (a value indicating the minimally-acceptable reliability) [42, 43] and the validity assessment should yield similar patterns between the two languages. We evaluated the internal-consistency reliability with Cronbach’s α; test-retest reliability with intraclass correlation coefficient (ICC); known-group validity by comparing mean scores based on the relationship with the PWD and the stage of dementia (these two variables have been known to demonstrate contrasting levels of PDG in the literature) [8, 10, 11, 38]; and construct validity by correlation with ZBI and CES-D using Spearman’s correlation-coefficient (ρ). All analyses were performed with STATA software version 13.

## Results

The demographic information of the 394 participants is shown in Table 1. The participants comprised multiple ethnicities, with a predominance of Chinese (86.6%). Spousal caregivers constituted 13.7%, with the rest being children caregivers. Participants who completed the Chinese version were slightly older (*p* = 0.015), less educated (*p* < 0.001) and less likely to be primary caregivers (*p* = 0.045).

**Table 1:** Demographic information of the caregivers and the persons with dementia (*n* = 394)

Variable	Overall sample (*n* = 394)	Chinese version (*n* = 103)	English version (*n* = 291)	*P* value^a^
Variables related to caregivers				
Age, mean (SD)	53.0 (10.7)	55.2 (9.3)	52.2 (11.0)	**0.015**
Female gender, *n* (%)	236 (59.9)	61 (59.2)	175 (60.1)	0.871
Ethnic, *n* (%)				**< 0.001**
Chinese	341 (86.6)	103 (100)	238 (81.8)	
Malay	25 (6.3)	0 (0)	25 (8.6)	
Indian	18 (4.6)	0 (0)	18 (6.2)	
Others	10 (2.5)	0 (0)	10 (3.4)	
Marital status, *n* (%)				0.630
Married	271 (68.8)	74 (71.8)	197 (67.7)	
Single	94 (23.9)	21 (20.4)	73 (25.1)	
Widowed/Divorced/Separated	29 (7.3)	8 (7.8)	21 (7.2)	
Employment status, *n* (%)				0.059
Not working	123 (31.2)	40 (38.8)	83 (28.5)	
Working part-time	52 (13.2)	16 (15.5)	36 (12.4)	
Working full-time	219 (55.6)	47 (45.6)	172 (59.1)	
Highest education, *n* (%)				**< 0.001**
Primary or no formal education	41 (10.4)	33 (32.0)	8 (2.8)	
Secondary	228 (57.9)	54 (52.4)	174 (59.8)	
Tertiary	125 (31.7)	16 (15.5)	108 (37.5)	
Relationship with PWD, *n* (%)				0.104
Child	340 (86.3)	84 (81.6)	256 (88.0)	
Spouse	54 (13.7)	19 (18.5)	35 (12.0)	
Staying with PWD, *n* (%)	264 (67.0)	70 (68.0)	194 (66.7)	0.810
Duration of caregiving in years, mean (SD)	6.8 (6.7)	7.2 (6.6)	6.6 (6.7)	0.514
Frequency of contact with PWD, *n* (%)				0.436
Daily, for at least 4 h a day	211 (53.6)	59 (57.3)	152 (52.2)	
Daily, but less than 4 h a day	79 (20.0)	15 (14.6)	64 (22.0)	
At least once a week	84 (21.3)	24 (23.3)	60 (20.6)	
Less than once a week	20 (5.1)	5 (4.9)	15 (5.2)	
Primary caregiving role, *n* (%)	279 (70.8)	65 (63.1)	214 (73.5)	**0.045**
ZBI score, mean (SD)	34.8 (16.8)	33.5 (14.1)	35.3 (17.6)	0.331
CES-D score, mean (SD)	15.7 (11.0)	17.4 (11.5)	15.1 (10.8)	0.064
Variables related to PWD				
Age, mean (SD)	79.5 (8.2)	79.7 (8.3)	79.4 (8.1)	0.779
Female gender, *n* (%)	278 (70.6)	72 (69.9)	206 (70.8)	0.865
Duration of dementia diagnosis in years, mean (SD)	4.5 (3.5)	4.3 (3.3)	4.5 (3.5)	0.500
Stage of dementia, *n* (%)				0.626
Mild	62 (15.7)	14 (13.6)	48 (16.5)	
Moderate	163 (41.4)	41 (39.8)	122 (41.9)	
Severe	169(42.9)	48 (46.6)	121 (41.6)	

SD, standard deviation; PWD, persons with dementia; ZBI, Zarit Burden Interview; CES-D; Centre for Epidemiologic Studies Depression Scale

^a^Test of difference between Chinese and English versions: chi-square test for categorical variables and two-sample T-test for continuous variables. Bold-faced *p* values are ≤0.05

**Fig. 1:**
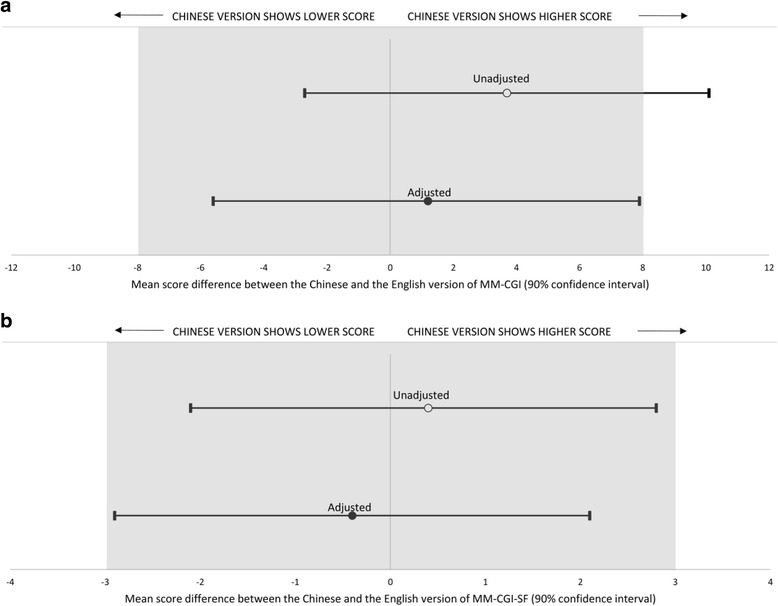
Mean score-difference between the Chinese and the English version of **a** Marwit-Meuser Caregiver Grief Inventory (MM-CGI); and **b** Marwit-Meuser Caregiver Grief Inventory-Short Form (MM-CGI-SF). The greyed areas indicate the margins of equivalence, based on approximately 5% of the reported average scores from the original publications [16, 17]. MM-CGI has a maximum score of 250, while MM-CGI-SF has a maximum score of 90. The adjusted models accounted for relationship with PWD, stage of dementia, and baseline variables which were significantly different between the two languages (caregiver’s age, ethnicity, education and role as primary caregiver)

The Chinese and English versions showed similar mean scores: 144.1 (SD 28.0) and 140.5 (SD 35.6) respectively for MM-CGI; and 51.9 (SD 10.6) and 51.5 (SD 13.5) respectively for MM-CGI-SF. The mean score-difference between the Chinese and English versions are illustrated in Fig. 1. In the adjusted model, the score-difference was 1.2 (90% CI -5.6 to 7.9) for MM-CGI and − 0.4 (90% CI -2.9 to 2.1) for MM-CGI-SF. The 90% CI for the adjusted score-differences fell within our predefined margins of equivalence (±8 for MM-CGI and ± 3 for MM-CGI-SF) and indicated equivalence of the scores. The adjusted score-differences for the subscales of MM-CGI and MM-CGI-SF are presented in Table 2. Although the subscales did not meet the 5% equivalence margin, all of them had the 90% CI falling within the less-stringent 10% margin which indicated possible equivalence.

**Table 2:** Score-difference between the language versions of MM-CGI subscales and MM-CGI-SF subscales

Subscales of MM-CGI and MM-CGI-SF	Mean scores (SD)		Equivalence margin (5%)^a^	Equivalence margin (10%)^b^	Difference between Chinese and English version (90% CI)	
	Chinese	English			Unadjusted	Adjusted^c^
MM-CGI						
*Personal Sacrifice Burden* subscale	54.8 (12.9)	53.3 (14.5)	±3	±6	1.5 (−1.2 to 4.2)	1.0 (−1.8 to 3.8)
*Heartfelt Sadness and Longing* subscale	44.0 (8.8)	43.3 (11.9)	±2	±4	0.8 (− 1.4 to 2.9)	−0.5 (−2.7 to 1.8)
*Worry and Felt Isolation* subscale	45.3 (8.9)	43.9 (11.4)	±2	±4	1.4 (−0.6 to 3.5)	0.7 (−1.5 to 2.9)
MM-CGI-SF						
*Personal Sacrifice Burden* subscale	18.7 (4.7)	18.2 (5.3)	±1	±2	0.4 (−0.5 to 1.4)	0.3 (− 0.7 to 1.4)
*Heartfelt Sadness and Longing* subscale	17.5 (3.6)	17.7 (4.8)	±1	±2	−0.2 (−1.1 to 0.6)	− 0.6 (− 1.5 to 0.3)
*Worry and Felt Isolation* subscale	15.7 (4.0)	15.6 (4.9)	±1	±2	0.2 (−0.7 to 1.1)	−0.2 (−1.1 to 0.8)

MM-CGI, Marwit-Meuser Caregiver Grief Inventory; MM-CGI-SF, Marwit-Meuser Caregiver Grief Inventory-Short Form; SD, standard deviation; CI, confidence interval

^a^The pre-defined margins of equivalence represented approximately 5% of the reported average subscale scores from the original publications [16, 17]

^b^The pre-defined margins of equivalence represented approximately 10% of the reported average subscale scores from the original publications [16, 17]

^c^The adjusted models accounted for relationship with PWD, stage of dementia, and baseline variables which were significantly different between the two languages (caregiver’s age, ethnicity, education and role as primary caregiver)

**Fig. 2:**
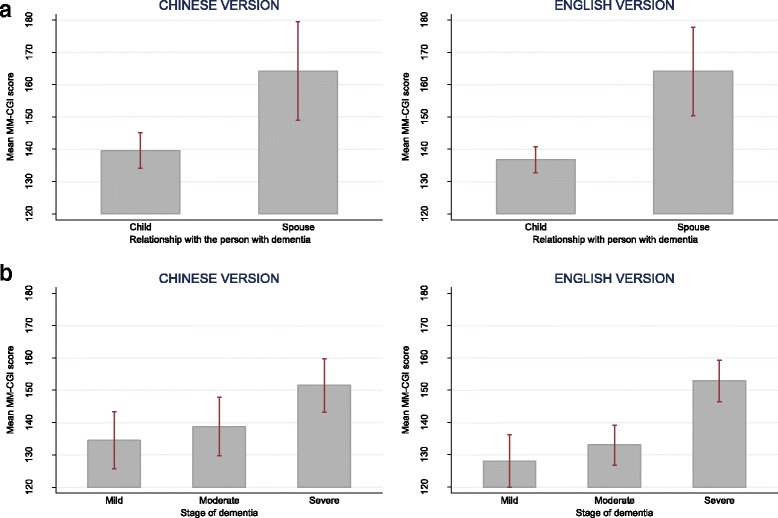
Comparison of the mean scores of Marwit-Meuser Caregiver Grief Inventory (MM-CGI) between groups which are known to differ in pre-death grief level, in the assessment of known-group validity. Figure **a** shows the mean scores of MM-CGI for child and spousal caregivers. Figure **b** shows the mean scores of MM-CGI at various stages of dementia. The vertical lines of error bar indicate the 95% confidence interval of the scores

**Fig. 3:**
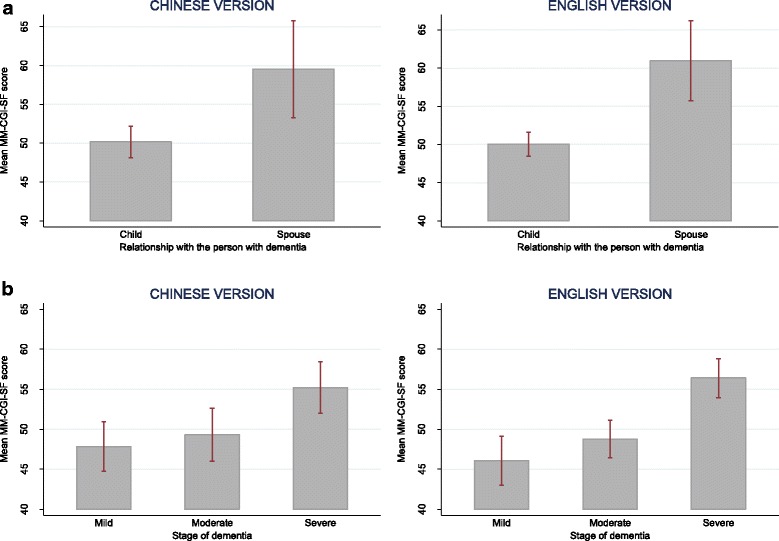
Comparison of the mean scores of Marwit-Meuser Caregiver Grief Inventory-Short Form (MM-CGI-SF) between groups which are known to differ in pre-death grief level, in the assessment of known-group validity. Figure **a** shows the mean scores of MM-CGI-SF for child and spousal caregivers. Figure **b** shows the mean scores of MM-CGI-SF at various stages of dementia. The vertical lines of error bar indicate the 95% confidence interval of the scores

The two language versions demonstrated similar patterns in internal consistency reliability, test-retest reliability, known-group validity and construct validity. The internal consistency and test-retest reliability of the two versions are presented in Table 3, with the 95% CI of all the reliability indices ≥0.70. For known-group validity, both versions showed similar characteristics of higher MM-CGI scores in spouses and in later stage of dementia (see Fig. 2 for MM-CGI; and Fig. 3 for MM-CGI-SF). For construct validity (Table 4), both language versions correlated strongly (Spearman’s *ρ*> 0.50) [44] with the total scores of caregiver burden (ZBI) and depression (CES-D) (*ρ*=0.61–0.77); and less strongly (*ρ*≤0.50) [44] with *Finance* subscale of ZBI, *Positive Affect* subscale of CES-D and *Interpersonal Problems* subscale of CES-D (*ρ*=0.16–0.49).

**Table 3:** Comparison of the pattern in reliability indices between the Chinese and English versions of Marwit-Meuser Caregiver Grief Inventory

	Chinese version (*n* = 103)	English version (*n* = 291)
Internal consistency reliability, Cronbach’s α (95% CI)^a^		
MM-CGI	0.96 (0.95–0.97)	0.98 (0.97–0.98)
MM-CGI-SF	0.91 (0.87–0.94)	0.94 (0.93–0.95)
Test-retest reliability, ICC (95% CI)^a^		
MM-CGI	0.83 (0.76–0.91)	0.90 (0.86–0.93)
MM-CGI-SF	0.79 (0.70–0.88)	0.88 (0.84–0.92)

*MM-CGI* Marwit-Meuser Caregiver Grief Inventory, *MM-CGI-SF* Marwit-Meuser Caregiver Grief Inventory-Short Form, *CI* confidence interval, *ICC* intraclass correlation coefficient

^a^The 95% CI of the reliability indices were estimated using the bootstrap method

**Table 4:** Comparison of correlation pattern among various scales, using Spearman’s correlation coefficient ρ (*n* = 394)^a^

	MM-CGI		MM-CGI-SF	
	Chinese	English	Chinese	English
ZBI total scale	0.76^e^	0.77^e^	0.69^e^	0.73^e^
* Burden in the Relationship* subscale	0.72	0.69	0.68	0.68
* Emotional Well-being* subscale	0.64	0.73	0.60	0.69
* Social and Family Life* subscale	0.65	0.71	0.59	0.68
* Loss of Control* subscale	0.64	0.68	0.55	0.63
* Finances* subscale	0.35^f^	0.49^f^	0.30^b, f^	0.45^f^
CES-D total scale	0.72^e^	0.77^e^	0.61^e^	0.75^e^
* Depressed Affect* subscale	0.74	0.75	0.65	0.71
* Somatic Symptoms* subscale	0.75	0.75	0.67	0.72
* Interpersonal Problems* subscale	0.36^f^	0.48^f^	0.33^f^	0.47^f^
* Positive Affect* subscale	−0.27^c, f^	− 0.43^f^	−0.16^d, f^	− 0.39^f^

*MM-CGI* Marwit-Meuser Caregiver Grief Inventory, *MM-CGI-SF* Marwit-Meuser Caregiver Grief Inventory-Short Form, *ZBI* Zarit Burden Interview, *CES-D* Centre for Epidemiologic Studies Depression Scale

^a^All the unmarked correlations had *p* < 0.001

^b^*p* = 0.002

^c^*p* = 0.006

^d^*p* = 0.115

^e^Similar to the original studies [16, 17], MM-CGI and MM-CGI-SF correlated strongly (ρ> 0.50) [44] with caregiver burden and depression scales because they measure related, though discriminable, phenomena in caregiving

^f^MM-CGI and MM-CGI-SF correlated less strongly (*ρ*≤0.50) [44] with *Finance* subscale of ZBI, *Positive Affect* subscale of CES-D and *Interpersonal Problems* subscale of CES-D, because pre-death grief is expected to differ from constructs such as financial difficulties, positive feelings, or the feeling that others are being critical

## Discussion

This is the first study to produce the Mandarin-Chinese version of MM-CGI and MM-CGI-SF, and compare their psychometric properties to the original English version. It was possible to conduct such a study because Singapore is among the few countries in the world where both the Chinese and English languages are used as commonly. We demonstrated the equivalence of total scores between the Chinese and English versions, and also demonstrated possible equivalence (at ±10% margins) in the subscale scores even though the sample size in the Chinese version (*n* = 103) was limited and may not have had sufficient power to establish equivalence using the more stringent ±5% margins. Additionally, we showed that the Chinese and English versions performed similarly in terms of internal consistency reliability, test-retest reliability, known-group validity and construct validity.

The findings from this study have several implications. With the availability of the Mandarin-Chinese version of MM-CGI, we can now expand the reach of PDG detection to family caregivers who use Mandarin-Chinese as their primary language. This is noteworthy as the Mandarin-Chinese language is used by approximately one-sixth of the world’s population [18], and Chinese-speaking populations have among the largest number of people living with dementia [1]. Detection of PDG provides an added dimension to supporting the emotional needs of family caregivers who are often the primary persons that provide most of the informal care to PWD [45] and thus can benefit from appropriate interventions for PDG.

The availability of the Mandarin-Chinese MM-CGI opens the way for further research to evaluate the efficacy of PDG interventions in dementia caregiving. To date, no intervention-studies have been conducted in the Chinese-speaking populations to address those with high PDG, possibly hindered by the lack of a relevant Chinese scale to assess PDG. Although prior studies have demonstrated the benefits of PDG interventions [7, 46, 47], they were primarily conducted in Caucasian populations. We remain uncertain whether PDG interventions are equally effective in the Chinese community, and whether there is a need to adapt the interventions to ensure that they can be culturally-relevant and sensitive to Chinese caregivers. In designing future intervention-studies for the Chinese community, researchers may possibly take reference from prior literature in this area [46–49] and include some of the commonly employed strategies such as: (1) encouraging caregivers to tell the story of the PWD through which they are helped to identify and process the painful emotions associated with the loss; (2) normalizing their emotions related to the loss and findings ways to remain connected with other caregivers; (3) educating caregivers on new ways to remain connected to the PWD such as through spiritual practices, celebrations, humor, life review and therapeutic touch; and (4) focusing on anticipatory grief and preparing the caregivers for potential future losses.

Measurement equivalence between the Chinese and English MM-CGI attests to the cross-cultural compatibility of the scale. Even though the Chinese and English languages are vastly different in their writing system (logogram- versus alphabet-based) and sentence structure, it was still possible to establish the measurement equivalence of the two versions. This supports the universal experience of PDG in caregivers of PWD [8, 38], and affirms the applicability of the PDG construct in the Chinese population. Indirectly, the measurement equivalence also provides evidence of the linguistic and cognitive equivalence between the Chinese and English versions [50]. It lends credence to the qualitative methods that were used to translate the Chinese version in this study.

Establishing measurement equivalence lays the ground for future research on the cross-cultural aspects of PDG in dementia caregiving. Although prior literature has suggested the influence of culture on the expression of grief [21, 38], we have limited understanding to date on how the characteristics of PDG vary across ethnic groups to affect dementia caregiving. It is now possible to use MM-CGI as a valid measure to compare PDG between the English- and Chinese-speaking populations because differences in PDG scores between the populations can be attributed to true variations in cultures per se, excluding scale language as the cause of differences. This is pertinent in the light of recent evidence demonstrating the influence of scale language on individuals’ responses to culture-related questionnaires [20].

Finally, evidence of equivalence justifies the pooling of data from the two language versions to improve statistical power and representativeness of future research on PDG. This can be especially relevant in multi-ethnic populations such as ours where the use of vernacular prevails in different subgroups of the population. Using this study as an illustration, as seen in Table 1, the English version captured a subpopulation which was younger and more educated, while the Chinese version comprised those who were older and less educated. Combining data from both language versions is thereby more representative of the overall population of caregivers.

Some limitations of the study should be noted. First, the participants were recruited from tertiary dementia services and may not fully represent those in the community. However, this is less likely a problem considering that most of the PWD in Singapore still receive their dementia care from tertiary centers, and the two recruitment centres in this study are the only two dementia services that serve the population in the North-East of Singapore. Second, the proportion of spousal caregivers in this study were much lower than that of the children caregivers (Table 1). Although it is possible that spousal caregivers may have been under-represented, this is less likely considering our proportion of spousal caregivers (13.7%) were not much different from the 16.0% reported in a separate study based on nationally representative samples [51]. Third, we had a smaller sample size for participants who completed the Chinese version of MM-CGI (*n* = 103). At the point of recruitment, our participants were given the choice to complete either the Chinese MM-CGI (from the current study) or the English MM-CGI (from our separate study [8]). Only about one-third of our participants chose to complete the Chinese version of MM-CGI and these participants were more likely to be older and less educated. This is not inconsistent with what we would have expected of the population in Singapore [52] – the younger population is typically more educated and bilingual (competent in English and Chinese), and has a preference for the English language; while the older population is less educated and usually more competent in Chinese than English. However, the smaller sample size in the Chinese version (*n* = 103) resulted in some limitations in our analyses. Ideally, we would have evaluated the factor structure of Chinese MM-CGI. However, such analysis is less recommended when the sample size is around 100 or less because it may produce improper solutions and unstable parameter estimates [53]. Fourth, as the scales were self-administered, it is possible that caregivers with lower literacy were under-represented. Fifth, the stage of dementia was assessed only based on self-reports by family caregivers, which may lack the precision of other staging instruments that also encompass objective assessments by the healthcare professionals. Sixth, our definitions on the equivalence-margins required some subjectivity in deciding on the values that would be small enough to reasonably claim equivalence, as there were no previous studies to provide guidance. Regardless, we have stated the rationale for our choice of the equivalence-margins to allow readers to judge whether these values are reasonable. Last, the MM-CGI-SF in this study was administered as part of MM-CGI and it might have different properties had it been administered separately on its own.

## Conclusion

In summary, this study has produced the Mandarin-Chinese version of MM-CGI and demonstrated its measurement equivalence, as compared to the English version, in detecting PDG. It opens the way for PDG assessment and intervention in Chinese-speaking caregivers, as well as cross-cultural research on PDG in dementia caregiving.

## Additional file

Additional file 1: The final version of the Chinese Marwit-Meuser Caregiver Grief Inventory. (DOCX 1019 kb)

## Abbreviations

CES-D: Centre for Epidemiologic Studies Depression Scale
CI: confidence interval
DSM-5: the fifth edition of Diagnostic and Statistical Manual of Mental Disorders
DSM-III-R: the revised third edition of Diagnostic and Statistical Manual of Mental Disorders
ICC: intraclass correlation coefficient
ISPOR: the International Society for Pharmacoeconomics and Outcomes Research
MM-CGI: Marwit-Meuser Caregiver Grief Inventory
MM-CGI-SF: Marwit-Meuser Caregiver Grief Inventory-Short Form
PDG: Pre-death grief
PWD: Persons with dementia
SD: Standard deviation
ZBI: Zarit Burden Interview
