# Supplementary material for: Detecting pre-death grief in family caregivers of persons with dementia: measurement equivalence of the Mandarin-Chinese version of Marwit-Meuser caregiver grief inventory
Source: BMC Geriatr. 2018 May 11;18:114. doi: 10.1186/s12877-018-0804-5 (PMC5948857; doi:10.1186/s12877-018-0804-5)
Supplement: Supplementary file 1 — The final version of the Chinese Marwit-Meuser Caregiver Grief Inventory. (DOCX 1019 kb) [file 12877_2018_804_MOESM1_ESM.docx]

**ADDITIONAL FILES**

Additional file 1. The final version of the Chinese Marwit-Meuser Caregiver Grief Inventory

**Note:** Readers can refer to the following websites for a copy of the English Marwit-Meuser Caregiver Grief Inventory:

1. <http://knightadrc.wustl.edu/About_Us/PDFs/MM-CGI-50%20Full%20Version.pdf>
2. <https://academic.oup.com/gerontologist/article/58/2/e150/3868411>
